# A Perspective on Mg/Al Laminated Metal Composites

**DOI:** 10.3390/ma19153345

**Published:** 2026-08-06

**Authors:** Binbin Li, Jun Tan

**Affiliations:** 1 National Engineering Research Center for Magnesium Alloy & National Key Laboratory of Advanced Casting Technologies, College of Materials Science and Engineering, Chongqing University, Chongqing 400044, China; lbbin522@163.com; 2 Mingyue Lake Laboratory, Chongqing 401122, China; 3 Lanxi Magnesium Materials Research Institute, Lanxi 321100, China

**Keywords:** Mg/Al, laminated metal composites, intermetallic compounds, interface

## Abstract

Mg/Al laminated metal composites (LMCs) combine the low density of Mg alloys with the good corrosion resistance and formability of Al alloys, showing great application potential in the lightweighting field. However, the difficulty in coordinating deformation between Mg and Al alloys, together with the inevitable formation of brittle Al-Mg intermetallic compounds (IMCs) at the interface when the two metals are directly bonded, severely deteriorates the mechanical properties of Mg/Al LMCs and hinders their transition toward practical engineering applications. This perspective systematically reviews the historical development and primary fabrication routes of Mg/Al LMCs, with a particular focus on the thermodynamics and kinetics of interfacial reactions, the shift from passive IMC suppression to active interfacial-phase design, and the emerging opportunities brought by artificial intelligence. Furthermore, the remaining challenges in process–interface–property correlations, multicomponent interlayer design, database construction for AI-driven design, and closed-loop intelligent manufacturing are identified. The aim is to guide future research through critical insights and perspectives, and to offer valuable references for the development of high-performance lightweight LMCs.

## 1. Background

As the lightest metal structural materials, magnesium (Mg) alloys have attracted much attention due to their low density, high specific strength [1], good electrical conductivity [2], high thermal conductivity [3] and excellent electromagnetic shielding properties [4]. However, drawbacks such as poor corrosion resistance and low deformability at room temperature severely hinder their further promotion in practical applications. As the most widely used lightweight metal materials to date, aluminum (Al) alloys possess characteristics such as wear resistance, corrosion resistance, and good formability. However, with the rapid development of modern industry, there is an urgent need for even lighter materials to meet the requirements of energy saving and environmental protection. Laminated metal composites (LMCs) combine two materials with complementary advantages, offering a new approach to address the challenge that a single material can hardly meet future application demands [5]. Examples include Al/Mg [6], Al/Cu [7,8], Ti/Steel LMCs [9], and Ti/Ni [10]. Rydz et al. [11]. investigated the plastic forming process of Al1050/AZ31/Al1050 LMCs during rolling, and observed the formation of intermetallic compounds (IMCs) in the bonding zone. Combined with industry data, this study theoretically demonstrated that after using these LMCs for structural components, a significant weight reduction of 60% can be achieved without compromising performance, highlighting its great engineering application value. The Ti/Ni composites produced by explosive welding and rolling achieved ultra-high shear bonding strength, emphasizing the importance of selecting optimal process parameters for difficult-to-deform materials [12]. The Al/Cu LMCs confirmed that slight hardness differences between dissimilar materials can promote uniform plastic deformation across the layers and enhance interfacial bonding strength [13]. Together, these systems indicate that the successful fabrication of LMCs requires the simultaneous control of processing parameters, deformation compatibility of the materials, and the microstructural evolution of the reaction layers.

**Figure 1:**
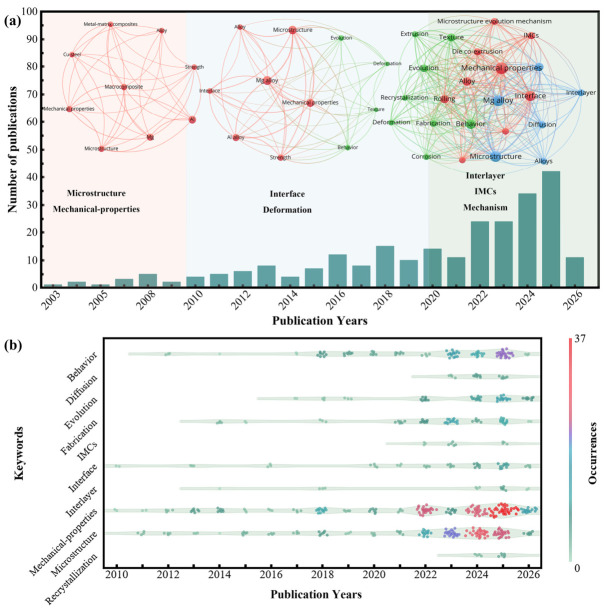
The number of publications retrieved from the Web of Science Core Collection using the topics “Mg/Al laminated metal composites”, “Mg/Al composite plates”, “Mg/Al composite sheets”, and “Mg/Al bimetal”, along with the research focuses of Mg/Al LMCs at different stages: (**a**) Number of publications. (**b**) Keywords corresponding to publication years.

Among these, Mg/Al LMCs integrate the low density and high specific strength of Mg alloys with the corrosion resistance and good formability of Al alloys, showing broad application prospects in aerospace, new energy vehicles, and other fields, and have become a focus of current research [14,15,16]. In 1996, Lesuer et al. [17] made early contributions to the development history, processing, and mechanical behavior of LMCs, during which the focus was primarily on the bonding of steel with other metals. As shown in Figure 1a, after 2000, research on Mg/Al LMCs gradually increased, marking the advent of an important era in the exploration of Mg/Al LMCs. In the early stages, researchers mainly focused on the mechanical properties and microstructure of Mg/Al LMCs. With further research, investigations into bonding strength, deformation behavior, and the interface began to deepen. It was found that IMCs formed at the interface severely degrade the performance of LMCs. Figure 1b presents the frequency of keywords corresponding to publication years. It can be observed that from 2010 to 2019, research on Mg/Al LMCs entered a phase of rapid development, with more processing methods being used to fabricate Mg/Al LMCs. The relationship between fabrication processes, the interface, and interfacial bonding strength gradually gained attention from researchers. In terms of keyword frequency, from 2010 to 2016, research in this field was still predominantly focused on mechanical properties and microstructure. Starting in 2017, the keyword ‘evolution’ newly emerged, while the frequency of the keyword ‘Behavior’ gradually increased, indicating that the field progressively shifted towards mechanistic studies of Mg/Al LMCs.

As shown in Figure 1b, after 2020, the frequencies of the keywords “Evolution,” “Behavior,” “Interface,” and “Fabrication” became more prominent, and an increasing number of preparation methods have been employed to investigate the interface and interfacial evolution behavior of Mg/Al LMCs, which is mainly attributable to advances in processing and microstructural characterization techniques. Compared with previous years, research on the mechanisms governing the performance of Mg/Al LMCs witnessed breakthrough development during this stage. In 2022 and 2023, the new keywords “IMCs” and “Diffusion” emerged, respectively. This is because, with deepening research, the influence of IMCs on the overall performance of LMCs has drawn increasing attention, making it necessary to study the mechanisms of their formation and evolution. The period from 2024 to 2026 has mainly focused on interfacial evolution behavior and the effects of the interlayer on the microstructure and mechanical properties of Mg/Al LMCs.

From the perspective of the different historical stages of Mg/Al LMCs development, this field has gradually evolved from initial studies on microstructure and mechanical properties to in-depth investigations into the mechanisms governing performance, particularly interfacial evolution and control behavior, which is highly beneficial for the research of high-performance lightweight LMCs. Meanwhile, environmental issues arising from conventional energy sources have imposed increasingly stringent requirements on energy conservation and emission reduction. Therefore, replacing traditional materials with lightweight and environmentally friendly materials can yield significant economic benefits while simultaneously saving energy. However, the room-temperature formability of magnesium alloys is extremely limited, and the difficulty in coordinating the deformation between Mg and Al alloys makes the fabrication of Mg/Al LMCs highly challenging. Moreover, the strong chemical affinity between Mg and Al readily induces the nucleation and rapid growth of Mg-Al IMCs, leading to the formation of an IMC layer at the interface, which severely deteriorates the material properties. These issues are insurmountable challenges that must be overcome in the future development of Mg/Al LMCs. This paper provides a comprehensive review of the development history of Mg/Al LMCs since the beginning of the 21st century, and visualizes the research focuses of different periods. Taking the two major challenges facing this field as the main thread, this review links preparation processes, thermodynamics and kinetics of dissimilar metal joining, interface modification, and potential development directions, aiming to provide insights for the development of high-performance LMCs from a unique perspective.

## 2. Fabrication of Mg/Al LMCs

A variety of processes have been employed to prepare Mg/Al LMCs. Based on the state of the materials before bonding, the bonding process can be classified into solid–solid bonding, solid–liquid bonding, and liquid–liquid bonding, among which the first two are the most widely used. Solid–solid bonding primarily relies on atomic diffusion, mechanical interlocking, and metallurgical bonding. Rolling, as a typical representative of solid–solid bonding, enables large-area bonding of Mg and Al alloys and is considered the most cost-effective method. Liu et al. [18] provided a comprehensive review on the preparation of Mg/Al LMCs by rolling. They pointed out that the rolling bonding process mainly involves matrix deformation and the formation of an interfacial reaction layer, with the latter having a decisive influence on the bonding strength of Mg/Al LMCs. In particular, when the thickness of the diffusion layer exceeds 5 μm, the bonding strength decreases sharply. Solid–liquid bonding primarily involves metallurgical bonding and diffusion bonding. Compound casting [19] typically involves pouring molten Mg alloy onto the surface of solid Al alloy to achieve bonding. Compared to other processes, this method allows the fabrication of components with complex structures. Welding can partially melt the metals, resulting in strong bonding at the interface, and also allows the addition of nanoparticles to form particle reinforcement at the interface, thereby enhancing bonding strength [20]. Explosive welding, as a representative technique, has been employed in research on Ti/Al [21], Ti/Ni [12], and other material systems. It forms a metallurgical bond through high velocity oblique collision, and the plastic flow and jetting effect disrupt surface oxides and create a wavy interface, making it suitable for joining metals with significantly different physical properties [22,23]. However, the interfacial jetting and localized melting can lead to the formation of IMCs in rapidly solidified zones, and subsequent hot rolling or heat treatment may accelerate their growth [24,25]. Therefore, it is essential to jointly optimize the welding parameters and the subsequent thermomechanical and heat treatment processes to control the interfacial morphology and mechanical properties. However, the solid–liquid bonding process is extremely difficult to control, and an excessively thick reaction layer can easily form at the interface, which represents the biggest obstacle to the widespread adoption of this process.

Although significant progress has been made in the research on the preparation processes of Mg/Al LMCs, the process parameters remain difficult to determine. In particular, large deformation processes subject Mg/Al LMCs to complex stresses during forming, which can affect surface quality or cause internal microcracks. Therefore, the optimal processing conditions are mostly determined through trial and error, and there is still a lack of practical methods to guide the forming process. More importantly, current research is still confined to small-scale laboratory samples. However, a recent study on the preparation of Al/Mg/Al LMCs via online heating rolling has achieved a breakthrough in terms of sample size, which is of great significance for promoting the transition of Mg/Al LMCs toward practical industrial applications.

## 3. Interfacial Thermodynamics and Kinetics in Dissimilar Metal Joining

**Figure 2:**
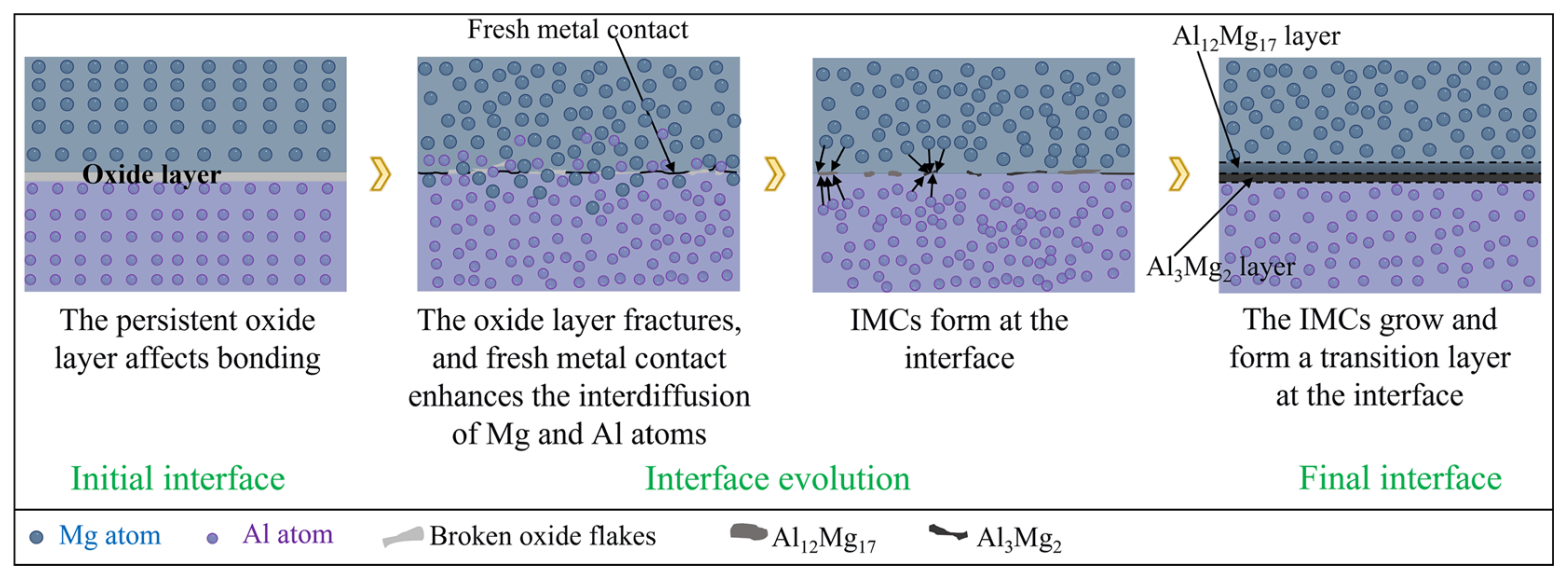
Schematic diagram of the fabrication and interfacial evolution of Mg/Al LMCs.

Due to the significant differences in physical and chemical properties, the bonding process between dissimilar metals typically results in the formation of an interfacial transition zone with a certain width. Importantly, the interface serves as a bridge for transferring external loads from one material to the other, and thus plays a critical role in determining the overall performance of the material. The formation of the interface is closely related to the mutual diffusion of atoms and the evolution of IMCs, as shown in Figure 2.

**Figure 3:**
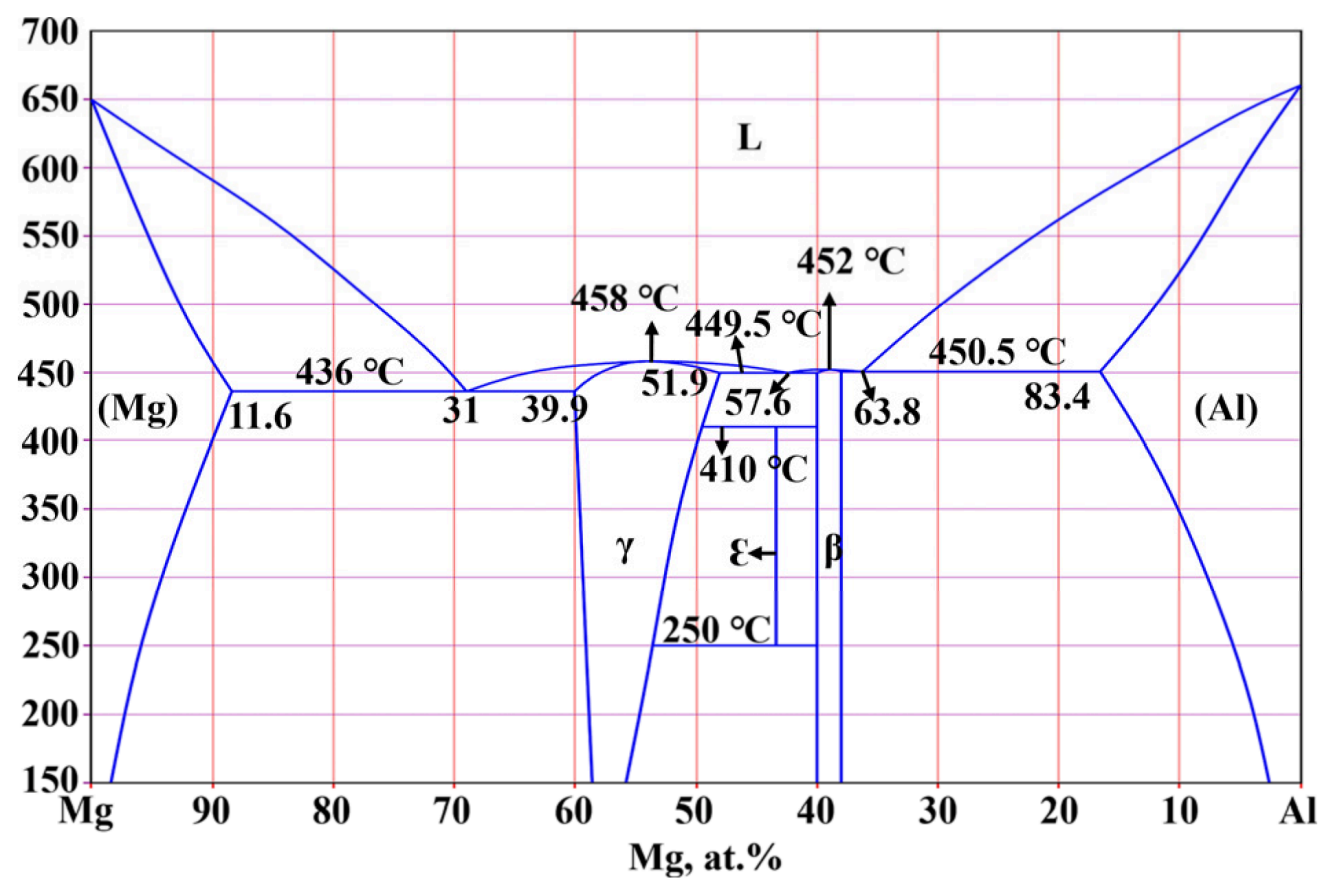
Mg-Al binary phase diagram adapted from Ref. [32].

According to the Mg-Al binary phase diagram (Figure 3), when Mg and Al are directly bonded, the γ (Mg_17_Al_12_) phase tends to form on the Mg-rich side, while the β (Mg_2_Al_3_) phase forms on the Al-rich side. In addition, variations in heat input caused by the preparation process and subsequent heat treatment can also lead to the formation of the non-equilibrium ε (Mg_23_Al_30_) phase [26,27]. The formation of IMCs is closely related to the rupture of the oxide film and atomic diffusion, and is governed by the combined constraints of thermodynamics and diffusion kinetics. The rupture of the oxide film on the metal surface exposes the fresh metal, and the sustained heat input intensifies atomic vibrations and promotes interdiffusion, which further leads to the formation of IMCs. Under the combined influence of thermodynamics and kinetics, different IMCs undergo nucleation and growth, gradually forming a continuous transition layer. During solid-state joining, the nucleation of IMCs relies primarily on atomic interdiffusion, whereas during solid–liquid joining, in addition to interdiffusion, the process also involves a stage of solidification nucleation. The growth of IMCs follows a parabolic law with respect to temperature and time, and increase in heat input leads to a thicker layer of IMCs, and the common IMCs at the Mg/Al interface include Al_3_Mg_2_ and Al_12_Mg_17_. At phase equilibrium, since the Gibbs free energy of Al_3_Mg_2_ is always higher than that of Al_12_Mg_17_, and solute atoms in the Mg matrix have a higher diffusion coefficient, Al_12_Mg_17_ preferentially forms [28,29]. However, during the growth stage, Al_3_Mg_2_ exhibits a much faster growth rate than Al_12_Mg_17_ because Al_3_Mg_2_ has a significantly higher diffusion coefficient at this stage [30]. Since both the Gibbs free energy and enthalpy of formation of the Al-Mg binary system are negative, the formation of Al-Mg IMCs will proceed spontaneously once certain conditions are met. This is the primary reason why the presence of IMCs at the interface is difficult to avoid. However, the differences in thermodynamic energy also offer researchers the possibility of controlling the types of interfacial IMCs. Gao et al. [31] investigated the joining of Mg-Li alloy to Al alloy. Thermodynamic calculations revealed that, owing to the competition between Mg and Li atoms arising from the chemical potential gradient, the types of IMCs formed at the interface transformed into Al_12_Mg_17_ and the ternary phase Al_2_MgLi. However, whether similar findings are applicable to other alloy systems remains to be further investigated. In-depth studies in this direction will open up new avenues for improving the types of interfacial products from the perspective of thermodynamics. Additionally, previous studies have focused on passively suppressing the formation of brittle IMCs. Currently, researchers have attempted to inhibit or prevent the formation of Al-Mg IMCs by adding interlayers, and have achieved promising results. Depending on the state of the materials prior to forming, interlayers can be classified into metallic interlayers and non-metallic interlayers. Metallic interlayers include pure metal interlayers (e.g., Cu foil, Zn foil, Ti foil, Ni foil), alloy interlayers, and multilayer interlayers combining different metals. However, few studies have conducted in-depth investigations into the nucleation and growth of IMCs at the interface, especially regarding the nucleation stage, making it difficult to provide practical guidance for the preparation of Mg/Al LMCs. Therefore, enhancing the understanding of the thermodynamic and growth kinetic mechanisms of IMC formation at the interface is conducive to actively introducing IMCs that are beneficial for enhancing interfacial bonding strength by modifying the composition of the added interlayer.

It is worth noting that the presence of IMCs is not entirely detrimental. Thin and discontinuous IMCs can impede crack propagation and thus be beneficial to the performance of Mg/Al LMCs [33]. Recent studies have pointed out that although Al/Mg/Al LMCs can still achieve relatively high bonding strength even with the presence of an Al-Mg IMCs layer at the interface, this may be attributed to the enhanced metallurgical bonding provided by IMCs with a thickness below 1 μm. However, the main reason for the decrease in bonding strength is considered to be the incompatible deformation between the Mg alloy and the Al alloy [6]. Unfortunately, once IMCs nucleate, they rapidly grow and thicken with the input of external energy, forming a continuous transition layer. An excessively thick IMC transition layer severely degrades material properties. Under external loading, these IMCs generate strong stress concentrations, leading to crack initiation, and subsequently accelerate crack propagation, hastening material failure [34].

The fabrication process and subsequent heat treatment are the main sources of heat input during interfacial evolution. However, systematic studies on the intrinsic relationship and mechanisms between processing parameters and interfacial evolution are still lacking. The formation of the interfacial transition layer generally involves IMCs nucleation and growth. Since the preparation of Mg/Al LMCs often requires sufficient heat to make the Mg layer deformable, IMCs easily undergo rapid growth once nucleated. Therefore, understanding the IMCs formation and evolution mechanisms and intervening in time to curb excessive growth are of great significance. These will help precisely control the interfacial structure and advance the development of Mg/Al LMCs.

## 4. IMCs from Suppression to Design

**Figure 4:**
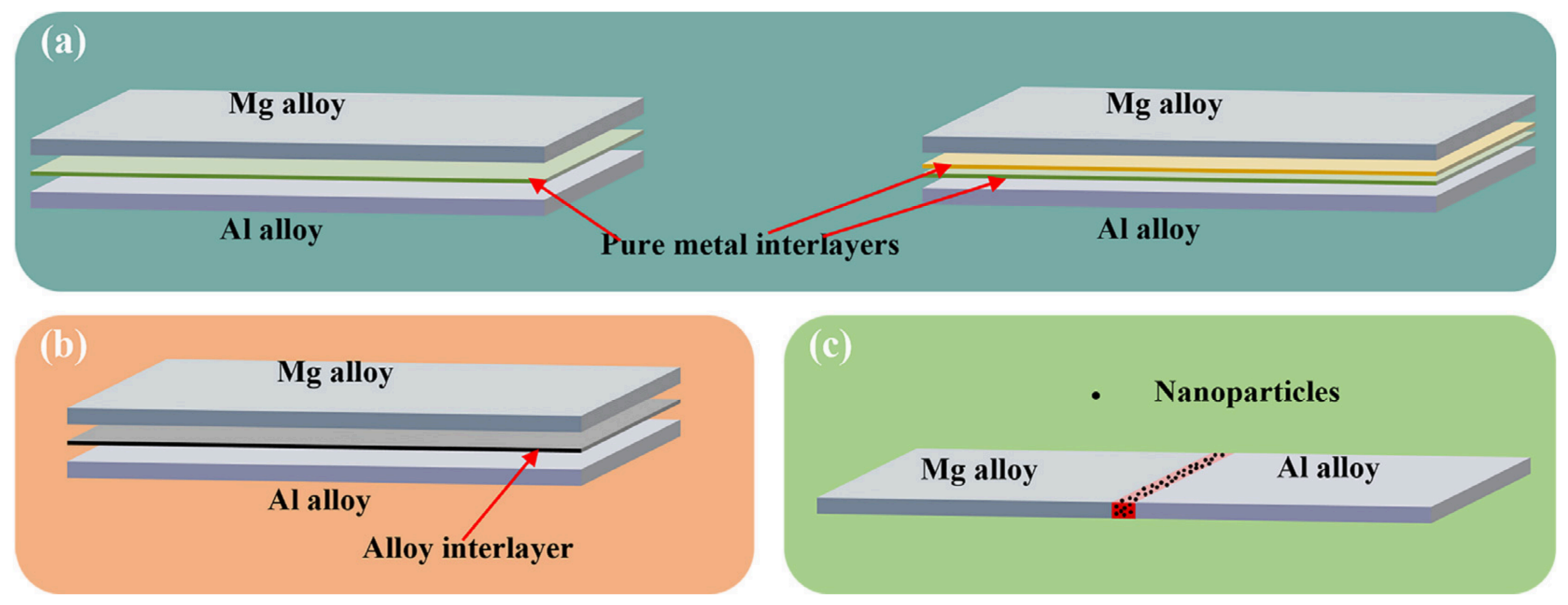
Mg/Al LMCs reinforced with different interlayers: (**a**) Pure metal interlayer and multilayer interlayers. (**b**) Alloy interlayer. (**c**) Nanoparticle reinforcement for joint strength.

Adding an interlayer can effectively regulate the interfacial IMCs, and the essence of its modification effect is to physically isolate the direct contact between Mg and Al alloys, thereby altering the type of interfacial reaction. Figure 4a–c shows a schematic diagram of Mg/Al LMCs fabricated with different interlayers. Most current studies focus on the addition of pure metal interlayers and the introduction of nanoparticles to increase joint strength. Compared with pure metal interlayers, the introduction of alloy interlayers involves the diffusion and bonding of multiple atoms. This makes the interfacial reaction process more complex. Therefore, fewer studies have been conducted on alloy interlayer addition. However, this also provides researchers with an opportunity to rationally design the IMCs at the interface. In addition, the types of IMCs formed are still constrained by the kind of interlayer added. For example, the addition of a pure Zn interlayer typically leads to the formation of MgZn_2_ and Al-Mg-Zn ternary IMCs [35,36], while a pure Ni interlayer leads to the formation of Al_3_Ni_2_, Mg_2_Ni and Mg_3_AlNi_2_ [37,38]. Studies have shown that even with the same interlayer material, different interlayer states and preparation processes can result in different IMC species. When pure Cu was used as a filler material to prepare Mg/Al LMCs via cold metal transfer welding [39], the main IMCs at the interface were AlCu, Al_2_Cu, Al_4_Cu_9_, and Cu_2_Mg. In another study, after cold spraying Cu powder onto the surface of a Mg alloy, Mg/Cu/Al LMCs were prepared by single pass hot rolling [40], and the main IMCs at the interface were AlCu, Al_4_Cu_9_, AlCuMg, and Al_5_Cu_6_Mg_2_.

It can also be observed that when a single interlayer is used for interfacial regulation, compounds related to that interlayer typically form at the interface. Although this approach can effectively suppress the formation of Al-Mg IMCs, it still has significant limitations. In addition, ultrasonic-assisted rolling of Mg/Cu/Al LMCs with varying ultrasonic amplitudes can lower the Gibbs free energy of the Mg-Al solid solution below that of Al_3_Mg_2_ IMCs, thermodynamically demonstrating the feasibility of suppressing the Mg-Al phase transformation solely through process modification [41]. Compared with suppressing Al-Mg IMCs using a single interlayer or process changes alone, a composite interlayer can simultaneously enhance bonding strength. Recently, a novel FeCoNiCrCu/Ni composite interlayer was used to prepare Mg/Al bimetals [42]. At the interface, the originally brittle IMC transition layer was transformed into a continuous Al*_x_*FeCoNiCrCu body-centered cubic structure layer, a residual high entropy alloy layer, and a solid solution layer containing a Mg_2_Ni eutectic structure on the Mg side. This multicomponent diffusion layer increased the shear strength of the Mg/Al bimetals by 132.05%, reaching 73.63 MPa. Therefore, future research on interface strengthening of Mg/Al LMCs can focus on designing suitable interlayers according to the service environment of the material to introduce novel diffusion layers, rather than merely suppressing the formation of brittle IMCs. Of course, whether the formation of a new diffusion layer introduces other adverse factors to the bonding interface must be considered at the initial design stage.

## 5. AI for Mg/Al LMCs

**Figure 5:**
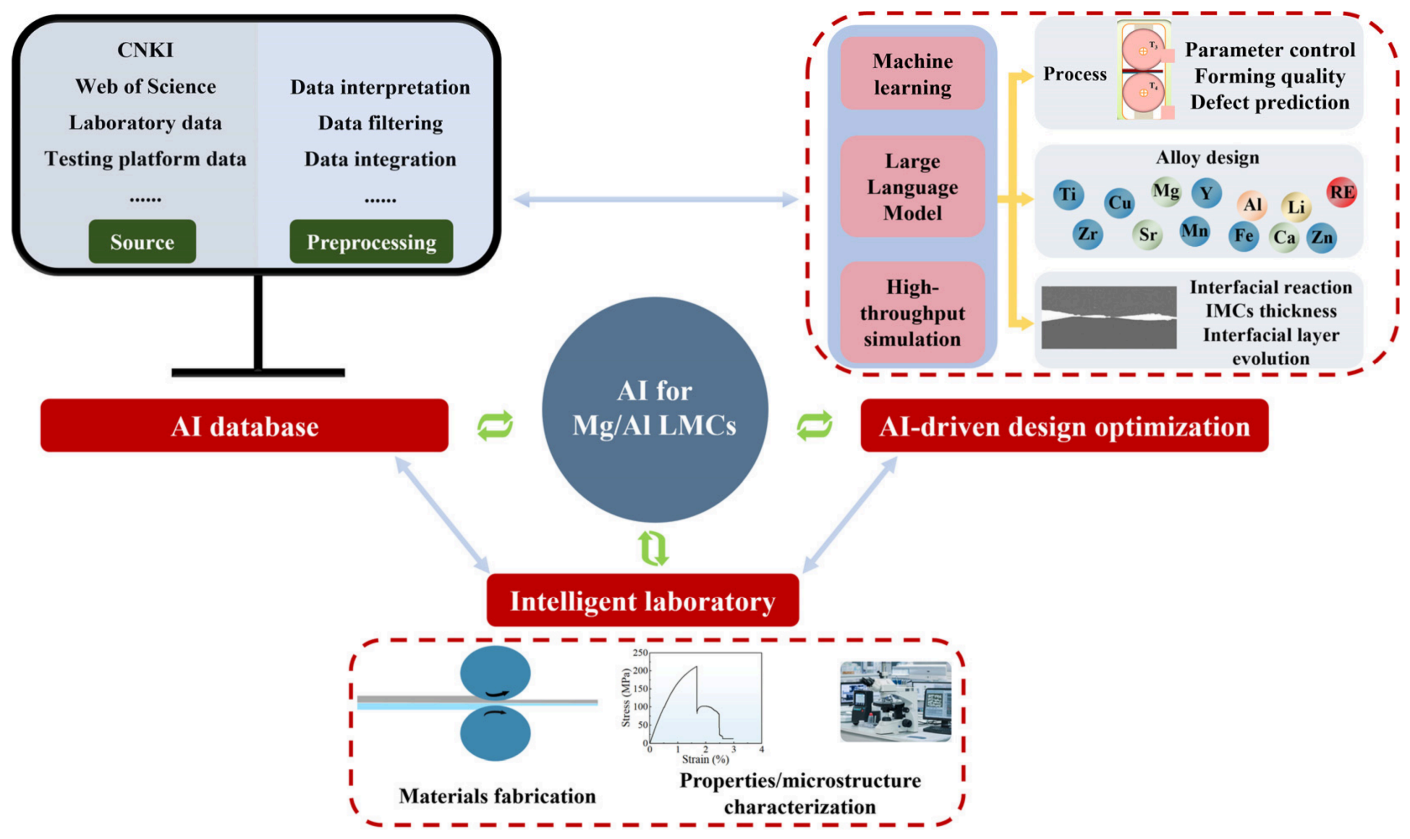
Schematic of the closed-loop system for AI-driven Mg/Al LMCs development: data integration, design and optimization, autonomous verification, and data application.

Looking back at the development of scientific research, it can be found that materials research has undergone a transition from early experimental trial-and-error to theoretical guidance and computational simulation. This transition has greatly promoted the development of new high-performance materials. However, a large number of research approaches cannot be carried out one by one. This has become a major obstacle to materials development. With the rapid advancement of artificial intelligence (AI), data-driven machine learning (ML) based on big data has emerged as a powerful method for accelerating the design of high-performance materials. It has already been applied to Mg alloy design [43,44,45,46]. This indicates that AI-assisted materials design and development is no longer a conceptual idea. Some researchers have made preliminary attempts to use ML to guide the fabrication of Mg/Al LMCs. Anne et al. [47] employed decision trees and a multimodal perception model in ML to interpret the effects of process parameters on the mechanical properties of Mg/Al LMCs coated with Ce powder. Zhai et al. [48] used a ML model to quantitatively map the process performance relationship for Mg/Al LMCs fabricated by friction stir welding. The results showed that appropriately reducing the rotational speed and increasing the welding speed within the processing window can effectively enhance the bonding strength of the welded joint. However, the mere application of ML is far from sufficient to demonstrate the full potential of AI-assisted materials design and development. The application of AI in the materials field should form a closed-loop system. This system includes data integration, AI-driven design and optimization, and autonomous verification by intelligent laboratories (IL). The verified data are then fed back into AI design. This process is illustrated in Figure 5.

At present, data scarcity remains the main bottleneck limiting the application of AI in advancing materials design [49]. Research on using AI to design self-improving closed-loop systems for autonomous manufacturing is becoming an urgent issue. This research also aims to accelerate the transition toward intelligent, data-driven production. In addition, in current research, Mg/Al LMCs are mostly fabricated using AZ series Mg alloy. It is well known that alloy composition has a significant impact on formability, and altering the chemical composition of Mg alloys can enhance their forming performance. For example, a Mg-4Li (wt.%) alloy exhibits no cracks on the surface even after 86% thickness reduction by cold rolling at room temperature [50], and a Mg-2Zn-0.8Gd (wt.%) alloy shows no edge cracks at a single-pass cold rolling reduction of 35% [51]. Therefore, the application of AI in Mg/Al LMCs has direct roles in designing process parameters and regulating the interface. In addition, AI can autonomously design Mg alloys with high strength and high formability. This further enhances the overall performance of the LMCs. It also greatly reduces the cost of experimental trial and error while improving work efficiency. In summary, with the advancement of science and technology, AI brings both challenges and opportunities for promoting the development of high-performance Mg/Al LMCs.

## 6. Conclusions

Mg/Al LMCs provide an effective route to combine the low density and high specific strength of Mg alloys with the corrosion resistance and formability of Al alloys. Studies have shown that their performance depends not only on the selected fabrication method, but more fundamentally on the deformation compatibility between dissimilar materials and the coupled evolution of interfacial reaction products. The formation and growth of interfacial reaction products are closely related to the fabrication process. Thin and discontinuous reaction layers can form metallurgical bonds, whereas thick and continuous interfacial reaction products tend to cause severe stress concentration and cracking tendencies, accelerating material failure. Therefore, the development of Mg/Al LMCs is shifting from empirically suppressing IMCs to actively designing interfacial-phase combinations and structures. Multicomponent or composite interlayers offer greater freedom in controlling diffusion paths and designing interfacial phases, but their long-term stability and potential adverse reactions must be evaluated under practical conditions.

Future research on Mg/Al LMCs can focus on the following priorities:

(1) Establishing quantitative process temperature time phase property diagrams.
(2) Combining phase diagram calculations and thermal diffusion models with in situ and multiscale characterization to investigate the nucleation process of early IMCs and the subsequent growth mechanisms of IMCs.
(3) Designing interlayers under explicit processing, thermal input, and manufacturability constraints.
(4) Fabricating and validating the performance of large-scale Mg/Al LMCs beyond laboratory-scale small-sample studies, which is expected to promote the transition of Mg/Al LMCs from experimental design to future engineering applications.
(5) Establishing standardized and traceable datasets for AI-driven design to shorten the research cycle for high-performance materials, and building intelligent experimental platforms to achieve a closed loop from AI design to validation.

## Data Availability

The original contributions presented in this study are included in the article. Further inquiries can be directed to the corresponding author.
